# Comparison of *Helicobacter bilis*-Associated Protein Expression in Huh7 Cells Harbouring HCV Replicon and in Replicon-Cured Cells

**DOI:** 10.1155/2012/501671

**Published:** 2012-07-30

**Authors:** Arinze S. Okoli, Mark J. Raftery, George L. Mendz

**Affiliations:** ^1^GenØK-Centre for Biosafety, Tromsø Science Park, 9294 Tromsø, Norway; ^2^School of Medical Sciences, The University of New South Wales, Sydney, NSW 2052, Australia; ^3^Bioanalytical Mass Spectrometry Facility, The University of New South Wales, Sydney, NSW 2052, Australia; ^4^School of Medicine, Sydney, The University of Notre Dame, New South Wales, Darlinghurst, NSW 2010, Australia

## Abstract

Hepatocellular carcinoma (HCC) is one of the leading causes of cancer-related deaths worldwide. Hepatitis B or C infections are the main causes of HCC with hepatitis C being the major risk factor for liver cancer in the developed countries. Recently, complications with bacteria of the genus *Helicobacter* have been associated with HCV-induced HCC. To further understand the mechanisms leading to the development of HCC in the presence of HCV and/or *Helicobacter* spp., investigation of the differential protein expression in Huh7 cells harbouring HCV-replicon, and replicon cured-Huh7 cells cocultured with *H. bilis* was done employing two-dimensional gel electrophoresis and mass spectrometry. In the transfected-Huh7 cells exposed to sublethal inoculum densities of *H. bilis*, 53 different proteins were identified comprising of 28 upregulated and 16 downregulated proteins including 9 potential protein isoforms; in the cured Huh7 cells, 45 different proteins were identified including 33 upregulated, 8 downregulated and, 9 potential protein isoforms. *H. bilis* affected the modulation of proteins involved in different pathways of Huh7-derived cells physiology including proteins involved in the progression from dysplasia to neoplasm. The result also indicated that the response of the Huh7-derived cells to the presence of *H. bilis* depended on whether or not HCV replicon was present.

## 1. Introduction

Hepatocellular carcinoma (HCC) is a malignancy of the liver caused by cirrhosis, the scarring of liver tissues. Cirrhotic liver results from chronic inflammation generally attributed to chronic and persistent infections of the liver by Hepatitis B virus (HBV) Hepatitis C virus (HCV), or alcohol abuse. Other carcinogens that have been associated with HCC include the *Aspergillus* aflatoxin B1, hemochromatosis, and fatty liver disease related to diabetes and obesity, but their frequencies of association with the liver cancer are lower than HBV or HCV.

Many of the chronic carriers of HBV or HCV do not develop cirrhotic liver, and only a subset of patients suffering from the viral-induced liver cirrhosis eventually progress to HCC, suggesting the existence of cofactors in hepatocarcinogenesis in the presence of HBV or HCV. For example, alcohol liver disease (ALD) has been documented as potentiating the development of the liver tumour in the presence of HBV or HCV [1], and syngergistic interactions between aflatoxin B1 and HBV have been reported in HCC [2]. In addition, information supports that coinfection with HBV and HCV increases the risk of HCC development over that with either viruses alone, and the increased risk is additive [3].

Recent information suggests the existence of bacteria cofactor in the progression of chronic viral hepatitis to cirrhosis and HCC. Bacteria DNA belonging to the *Helicobacter* genus have been increasingly identified in tissue specimen from patients suffering from HCV-induced HCC [4–7]. Further, in several HCV positive patients at different stages of the disease progression, *Helicobacter* DNA was found in 4.2% of the controls and 3.5% of the patients with noncirrhotic chronic hepatitis compared to 61–68% in cirrhotic liver and 90% in HCC tumoural tissue [8]. At different stages of the disease, the strength of association between the presence of *Helicobacter* DNA and the disease increased with severity of the cancer [8], suggesting that infections by *Helicobacter* spp. at some stage in the HCV-induced liver cirrhosis may contribute to the progression from dysplasia to neoplasia. The molecular mechanisms involved in the progression to cirrhosis and HCC in some patients suffering from HCV-induced hepatitis is still poorly understood, and the potential roles that *Helicobacter* spp. may play in HCC is largely unknown.

*Helicobacter* species cause persistent and chronic infections in their host cells where they induce strong inflammatory responses [9, 10]. Given the role played by chronic inflammation in malignant diseases in general, and specifically in cirrhosis and HCC, and considering reports of greater degree of hepatic damage [11] and higher incidence of cirrhosis [12] in dual infection of both HBV and HCV, or infection of either virus in a background of ALD or aflatoxin B1 intoxication, the coinfection of HCV and *Helicobacter* spp. may have a role in the development of liver malignancy. These coinfections may be one of the triggers required for the progression from cirrhosis to cancer in HCV-induced HCC. The association between HCC and *Helicobacter* spp. is further enforced by the finding that *H. hepaticus* induces chronic active hepatitis and HCC in A/JCr mice [13, 14], as well as the classification of *H. pylori* as a human carcinogen [15]. In this study, the effects of *H. bilis* on the proteome of Huh7 cells harbouring HCV replicon (transfected Huh7) and in replicon-cured Huh7 cells (cured Huh7 cells) were investigated.

*Helicobacter bilis* is a Gram-negative microaerophilic bacillus with urease, catalase, and oxidase activity, and a member of the enterohepatic *Helicobacter* species (EHS) that generally colonize the intestines, and livers of animals and birds. *Helicobacter bilis* colonizes the gall bladder, lower intestine and liver of mice where it causes chronic hepatitis and HCC [16, 17]. Human hepatoma Huh7 is a well-differentiated liver epithelial cell line that is used commonly in the *in vitro* studies of the liver and its associated diseases [18]. In this study, Huh7 cells not cocultured with *H. bilis* served as controls; thus, comparative analysis between the experimental and control cell population will reveal the effects of the bacteria, notwithstanding that malignant transformation is already present in the hepatoma cells. The Huh7 transfection with Nneo3–5B (RG) is stable and permits the replication of HCV subgenomic replicon. The cured Huh7 is the rescued transfected Huh7 by treatment with interferon alpha (IFN-*α*).

Global analyses of the proteome of cells grown under different conditions provide important information about the physiological processes that take place in the cells, the identification of new cancer biomarkers and disease-associated targets, and pathogenic processes. Proteomic analyses have been employed to study changes in protein expression related to carcinogenesis [19, 20]. In the present study, two-dimensional polyacrylamide gel electrophoresis was employed to analyse the global protein expression profiles of cytosolic proteins obtained from the two Huh7-derived cell lines cocultured with and without *H. bilis*. Identities of differentially expressed proteins were determined by tandem mass spectrophotometry. Additionally, real-time polymerase chain reaction was employed to determine the differential expression of known HCC-related genes by the Huh7 cell lines in response to *H. bilis*.

## 2. Materials and Methods

### 2.1. Growth Conditions

The transfected and cured Huh7 cells were kind donations from Professor Peter White laboratory, School of Biotechnology and Biomolecular Sciences, the University of New South Wales, Sydney, Australia. The cells were transformed following the method of Inoue et al., 2007 [21], and the cured cells by treating the transfected cells with IFN-*α* using the method of Ikeda et al., 2002 [22]. The cells were maintained at 37°C in Dulbecco's Modified Eagle Medium (DMEM), containing 25 mM D-glucose, and 4.0 mM of Glutamax (L-alanyl-L-glutamine) as a substitute for L-glutamine (Invitrogen, Mulgrave, Vic, Australia), and supplemented with 10% Neonatal Calf Serum (NCS) (Invitrogen; Australia). Prior to cocultures with *H. bilis*, cells were cultured in a 75 cm^2^ vented tissue culture flasks (Interpath Services; Caringbah, NSW, Australia) under an atmosphere of 5% CO_2_ until *ca.* 50% confluency, and adapted for 6 h to the coculture media, which consisted of 9.25 g/L Brain Heart Infusion (BHI) (Oxoid, Australia), 10% (v/v) Horse Serum (Oxoid, Heilderberg, Vic, Australia), 10% (v/v) NCS and 80% (v/v) DMEM.

*Helicobacter bilis* strain ATCC 51630 was grown in *Campylobacter* Selective Agar (CSA), consisting of Blood Agar Base No. 2 (Oxoid; Australia), supplemented with 5% (v/v) defibrinated horse blood (Oxoid, Australia). The media contained 2 *μ*g/mL fungizone (Bristol-Myers Squibb; Sydney, NSW, Australia), 0.32 *μ*g/mL polymixin B, 5 *μ*g/mL trimethoprim, and 10 *μ*g/mL vancomycin (Sigma; North Ryde, NSW, Australia). Bacterial cultures were incubated for 48 h at 37°C under the microaerobic conditions of 5% CO_2_, 5% O_2_ and 90% N_2_.

To study the effects of *H. bilis *on the transfected and cured Huh7 cells, bacteria were harvested from plates and grown for 6 h in the coculture media. Prior to cocultures with the human cells, the bacteria were washed in BHI broth and resuspended to a density of OD_600_ ~1.00. Appropriate volumes of *H. bilis* were inoculated into the semiconfluent hepatoma cell cultures at densities of upto 10^10^ cfu/mL. The cocultures were incubated at 37°C for 48 h under an atmosphere of 5% CO_2_, 5% O_2_, and 90% N_2_. Control cultures, consisting of hepatoma cells in coculture media without bacteria were grown under the same conditions. Comparison of the growth of the hepatoma cells between microaerobic conditions and normal atmosphere of 5% CO_2_, 19% O_2_, and 78% N_2_ was also carried out. After 48 h incubation, cell morphology was examined by inverted microscopy. The culture media was poured off from the vented flasks and cell monolayers were gently washed three times with sterile phosphate buffered saline (pH 7.6) to remove the bacteria from the cocultures containing *H. bilis*. To detach the cells, the flasks were flooded with 5 mL of Trypsin/EDTA (0.13%/0.03%), and incubated at 37°C in 5% CO_2_ for approximately 20 min or until the cells were detached. The live/dead status of the Huh7 cells was determined by trypan blue staining to assess cell viability, and the number of live cells was counted using an improved neubauer cytometer. Bacterial growth was measured at 48 h and the morphology and purity of the cultures were determined by phase-contrast microscopy. Bacteria were grown on CSA plates to examine the creamy characteristics.

### 2.2. Preparation of Protein Samples

To determine differential protein expression, the Huh7-derived cells were grown in coculture media under a microaerobic atmosphere at 37°C without bacteria or with 10^3^ cfu/mL *H. bilis*. After 48 h incubation, the transfected and cured Huh7 cells were detached, harvested by centrifugation at 1000 ×g for 25 min at 4°C, washed thrice with 30 mL 0.2 M ice cold sucrose, mixed by pipetting, and centrifuged again at 1000 ×g for 25 min at 4°C. The resulting cell pellet was collected, resuspended in 1 mL TSU buffer, and disrupted on ice by sonication with a Branson digital sonifier (Branson Ultrasonics Corporation; Danbury, CT, USA) at amplitude of 30% for 15 s at a 5 s pulse and 5 s delay between pulses. This was repeated 15 times, and resulting suspension was centrifuged at 14000 ×g for 20 min at 4°C to remove cell debris, the supernatant was collected and nucleic acids were removed by adding 10 *μ*L nuclease buffer and incubating for 20 min at 4°C. Aliquots of the protein cell-free extracts were stored at –80°C for a maximum of three months or until used for 2D-gel electrophoresis.

The protein concentration of cell-free extracts was estimated by the bicinchoninic acid assay employing a microtitre protocol (Pierce, Rockford, IL, USA). Optical densities were measured at 595 nm using a Beckman Du 7500 spectrophotometer to determine the absorbances of the copper complexes in both samples and standards. The protein concentration of each sample was calculated based on a calibration curve constructed with known concentrations of BSA.

### 2.3. Two-Dimensional Gel Electrophoresis and Image Analyses

Two-dimensional polyacrylamide gel electrophoresis (2D-PAGE) was performed as previously described [23] with some modifications. In the first dimension, an aliquot containing 150 *μ*g of protein was made up to a final volume of 250 *μ*L in freshly prepared rehydration buffer containing 8 M urea, 100 mM dithiothreitol (DTT), 65 mM 3-[(3-cholamidopropyl)-dimethyammonio]-1-propanesulfonate (CHAPS), 40 mM Tris-HCL, pH 8.0, and 10 *μ*L of pH 4–7 IPG buffer. Samples were centrifuged at 14000 ×g at 4°C for 20 min to clarify the supernatants and were loaded onto an 11 cm immobiline dry strip pH 4–7 (Bio-Rad; Regents Park, NSW, Australia) in an immobiline tray. Isoelectric focusing was performed at 14°C using the IsoelectrIQ^2^ (Proteome Systems; North Ryde, NSW, Australia), programmed at 300 V fast voltage ramp for 4 h, 10,000 V linear voltage ramp for 8 h, and 10,000 V fast/linear voltage ramp for 12 h, or until 120,000 Vh were reached. Following isoelectric focusing, strips were equilibrated in two buffers containing 6 M urea, 20% (v/v) glycerol, 2% (w/v) SDS, 375 mM Tris-HCl; the first with 130 mM DTT and the second with 135 mM iodoacetamide (IA).

In the second dimension, sodium dodecyl sulphate-polyacrylamide gel electrophoresis (SDS-PAGE) was performed on criterion system precast 12.5% acrylamide gels (Bio-Rad, Australia) at 14°C and 50 V for 1 h, followed by 64 mA for 2 h or until the bromphenol blue dye front reached the bottom of the gels. Gels were fixed separately in 100 mL of fixing solution (50% v/v methanol, 10% v/v acetic acid) with gentle shaking for a minimum of 0.5 h, stained employing a silver staining method [24], and imaged using a Umax PowerLook-1000 flatbed scanner (FujiFilm; Tokyo, Japan). For comparative gel-image analysis, data were acquired and analyzed using the Z3 software package (Compugen; Jamesburg, NJ, USA). Statistical analyses (Student's *t*-test, 95% confidence interval) were performed on three gels from each growth conditions (experimental versus control) to determine the differential spot intensities between both conditions. In the analyses, a gel from cells grown without bacteria served as the reference gel; master gels were compiled from three gels of each growth condition, and were compared to determine the relative intensities of each protein spot.

### 2.4. Mass Spectrometry Identification of Proteins

Protein spots showing two-fold or more differences in intensity between both experimental conditions were cut out of the gels and washed twice for 10 min in 200 *μ*L of 100 mM NH_4_HCO_3_, reduced at 37°C for 1 h with 50 *μ*L of 10 mM DTT, alkylated for 1 h in 50 *μ*L of 10 mM IA, washed for 10 min with 0.2 mL of 10 mM NH_4_HCO_3_, dehydrated in acetonitrile, and trypsin-digested with 10 ng/*μ*L of trypsin (Promega; Annandale, NSW, Australia). After digestion for 14 h at 37°C, peptides were extracted by washing the gel slice for 15 min with 25 *μ*L 1% formic acid, followed by dehydration in acetonitrile. Digests were then dried in vacuo, resuspended in 10 *μ*L 1% formic acid and separated by nano-LC using an Ultimate/Famos/Switchos system (LC Packings, Dionex; Lane Cove, NSW, Australia). Samples (5 *μ*L) were loaded on to a C18 precolumn (Micron; 500 *μ*m × 2 mm) with buffer A (98% H_2_O, 2% CH_3_CN, 0.1% formic acid) and eluted at 25 *μ*L/min. After a 4 min wash, the flow was switched into line with a C18 RP analytical column (PEPMAP; 75 lm × 15 cm) and eluted for 30 min using buffer A at 200 *μ*L/min. The nano-electrospray needle was positioned ~1 cm from the orifice of an API QStar Pulsar tandem mass spectrometer (ABI; Foster City, CA, USA). The QStar instrument was operated in information-dependent acquisition mode. A time-of-flight mass spectrometry survey scan was acquired (m/z 350–1700, 0.5 s), and the two largest precursors (counts >10) were selected sequentially by Q1 for tandem MS analysis (m/z 50–2000, 2.5 s). A processing script generated data suitable for submission to database search programs. Collision induced dissociation spectra were analysed using the Mascot MS/MS ion search engine (Matrix Sciences; Boston. MA, USA) with the following parameters: trypsin digestion allowing up to one missed cleavage, oxidation of methionine, peptide tolerance of 0.25 Da, and MS/MS tolerance of 0.2 Da. Searches were performed on the National Centre for Biotechnology Information nonredundant (NCBI nr) database.

### 2.5. Real Time Polymerase Chain Reaction (qRT-PCR)

Total RNA was extracted from transfected- and cured Huh7 cells using the TRIzol reagent (Invitrogen, Australia) according to the manufacturer's instructions. The optical density measured at 260 nm was employed to determine RNA concentrations; and RNA purity was verified by measuring the optical density ratios, OD_260_/OD_280_ and OD_260_/OD_230_ using a NanoDrop ND-1000 spectrophotometer (Biolab; Mulgrave, VIC, Australia). RNA samples with OD_260_/OD_280_ < 1.8 or OD_260_/OD_230_ < 1.9 were further purified by overnight ethanol precipitation at –20°C in 3 M sodium acetate (pH 5.2). Purified RNA pellets were washed once with 80% ethanol and resuspended in DEPC-H_2_O. RNA samples were stored at –80°C for three months or until used.

Specific primers were designed based on the sequences published in the Human Genome available on the NCBI database; and employing the primer3 algorithm [http://www.genome.wi.mit.edu/genome_software/other/ primer3.html]. The properties of the primers were: melting temperatures between 60–63°C, length 19–23 bp, G-C content 50–55%, and expected size of the product 200–210 bp. The primer sequences used in this study is available on request.

To study the differential expression of genes reported to be associated with HCC, total RNA extracted from the Huh7-derived cells exposed to *H. bilis* was reverse-transcribed to cDNA using SuperScript III First-strand SuperMix kit (Invitrogen, Australia). Quantitative real-time PCR analyses were performed in triplicate using a Corbett Research Rotor Gene RG-3000 thermal cycler (Corbett Life Science; Sydney, NSW, Australia), employing the SYBR GreenER qPCR universal supermix according to the manufacturer's instructions (Invitrogen, Australia). Each reaction was performed in an individual tube in a seventy-two tube strips, containing 12.5 *μ*L supermix, 1.0 *μ*L of 100 ng/*μ*L forward primer, 1.0 *μ*L of 100 ng/*μ*L reverse primer, 1.0 *μ*L of 100 ng/*μ*L of cDNA, and DEPC-treated water to a total volume of 25 *μ*L. As controls, reactions were also run in the absence of template cDNA to detect any contamination for each primer set. Conditions for the qRT-PCR were 2 min at 50°C, 10 min at 95°C and 40 cycles each consisting of 15 s at 95°C, and 40 s at 60°C, and acquiring flourescence at 76°C for 15 s. At the completion of the PCR run, the temperature was increased from 72°C to 95°C for 115 s; the flourescence was measured continuously to construct melting curves. The relative expression of each target gene was normalized to the glyceraldehyde 3-phosphate dehydrogenase (GAPDH) gene using the method described by [25]. Briefly, the crossing points (CP) for each target gene were normalized to the geometric mean CP of the house keeping gene employing the following expression:

(1) Ratio=(ETarget)Δct Target(Control-Sample)(EReference)Δct Reference(Control-Sample),

where *E* is the amplification efficiencies of target and reference genes, assumed in this study to be 2 for all genes [26], and Ct is the comparative threshold cycle. The control/sample values were obtained with template cDNA from transfected- and cured Huh7 cells without bacteria and those exposed to sublethal *H. bilis* density of 10^3^ cfu/mL.

## 3. Results and Discussion

### 3.1. Growth of Huh7-Derived Cell Lines in CoCultures with *H. bilis*

**Figure 1:**
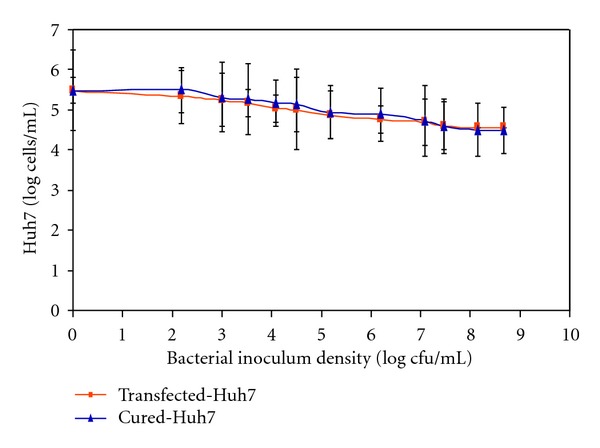
Growth of transfected- and cured Huh7 cells in cocultures with *H. bilis* at different inoculum densities. The initial number of Huh7 cells determined by the improved Neuber cytometer after staining with trypan blue was ca. 10^5.5^ cells/ml. The number of viable Huh7 cells counted after 48 h in cocultures with *H. bilis* are shown in the figure. The values represent the mean count of Huh7 cells from three independent experiments.

In the transfected- and cured Huh7 cells cocultured with *H. bilis*, hummingbird morphology was observed at bacterial densities of 10^3^ cfu/mL and higher. The results also revealed no significant (*P* > 0.05) decline in cell proliferation between the transfected and cured Huh7 cells (Figure 1), suggesting that neither the presence of the HCV-replicon nor its inactivation by IFN-*α* treatment affected differently the morphology and growth-response of the liver cells to the stress exerted by the presence of *H. bilis*. This phenomenon was similar to that observed in the parent Huh7 cells described previously (manuscript submitted elsewhere). This study did not investigate the response of the hepatoma cells to IFN-*α* treatment in the presence of *H. bilis* although it is acknowledged that the cured cells could also present the effects of IFN-*α*.

### 3.2. Differential Expression of Proteins by the Transfected- and Cured Huh7 Cell Lines in response to *H. bilis*

**Figure 2:**
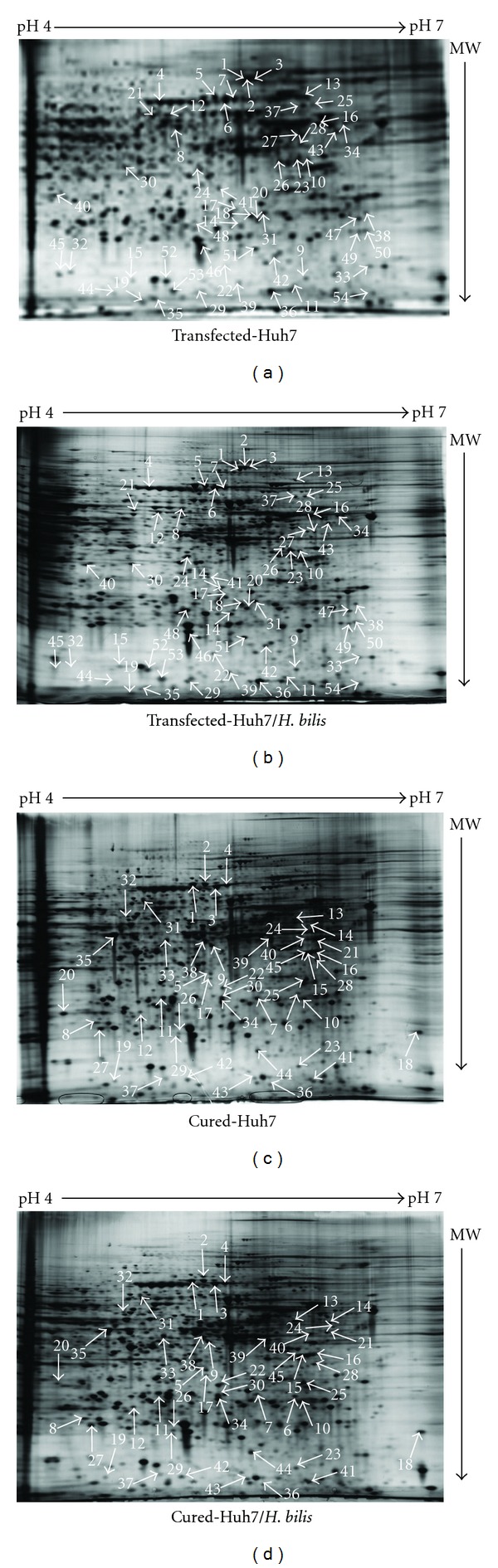
2D-PAGE gels showing spots of soluble proteins of transfected and cured Huh7 cells in the pI 4–7 range. Gels (a) and (b) correspond to proteins of transfected Huh7 cells, gels (c) and (d) correspond to proteins of cured Huh7 cells. Cells were incubated in the presence of an initial *H. bilis* inoculum of approximately 10^3^ cfu/mL. Protein spots in gels were visualised by silver staining. The spot intensities from triplicate gels of three independent experiments were determined using the Z3 computational software. Spots corresponding to proteins differentially expressed are indicated on the figure.

Total proteins from transfected- and cured Huh7 cells cultured in the presence and absence of *H. bilis* were extracted, purified, and separated in two dimensions employing a pH gradient of 4–7 for the first dimension, and an 11.5% SDS acrylamide gel in the second dimension. The intensities of protein spots from transfected- and cured Huh7 cells grown in the presence and absence of *H. bilis* were determined. Spots with differential intensities equal to or greater than 2-fold between cultures grown with and without bacteria were considered to be up or downregulated, and identified by LC-MS/MS.  Figure 2 shows four reference 2D-gels from each growth condition obtained from at least three independent experiments. In the transfected Huh7 cells exposed to sub-lethal inoculum densities of *H. bilis*, a total of 53 different proteins were identified comprising of 28 upregulated and 16 downregulated proteins, including 9 potential protein isoforms; in the cured Huh7 cells/*H. bilis* cocultures, 45 different proteins were differentially expressed including 33 upregulated, 8 downregulated, and 9 potential protein isoforms (Table 1). The potential protein isoforms consist of proteins that were identified in the same spots as highlighted in Table 1.

### 3.3. Biological Functions of the Modulated Proteins of Transfected- and Cured Huh7 Cell Lines in CoCultures with *H. bilis*

The identified proteins were related to several important biological functions, namely, regulation of cell proliferation and structure, metabolism and biosynthesis, protein translation and modification, regulation of transcription, stress response, signal transduction and transport, and tumour-related proteins (Table 1).

### 3.4. Metabolic Enzymes

Hepatocellular carcinoma often exhibits aberrant expression of metabolic enzymes. Various studies have shown perturbations of host metabolism during HCV infection [27, 28]. For example, [27] showed perturbations in glycolysis, the pentose phosphate pathway, and the citric acid cycle during *in vitro* HCV infection of Huh7.5 cells. In the present study, both transfected- and cured Huh7 cells modulated proteins of various metabolic pathways in response to *H. bilis*. The glycolytic enzymes enolase (ENO) and triosephosphate isomerase (TIM) were upregulated in the transfected Huh7 cells, and the cured Huh7 cell line also upregulated TIM, suggesting an upregulation of glycolysis by both types of cells in response to *H. bilis*. Upregulation of glycolysis would provide energy required by the hepatocytes to resist the stress exerted by *H. bilis*. Increased glycolysis is consistent with the upregulation of other enzymes involved in energy production observed in this study, namely, ATP5B and ATP5H in the transfected Huh7 cells; and ATP5B in the cured Huh7 cells. Similar upregulation of energy producing enzymes was observed in our previous study with the parent Huh7 cells to *H. bilis*, thus indicating a common response to *H. bilis* whether or not HCV replicon is present in the cell.

The enoyl coenzyme A hydratase 1, ECH1, participates in fatty acid, propanoate, and tryptophan metabolism. The enzyme was upregulated in the transfected Huh7 cells but was not found among the modulated proteins of the cured Huh7 cells in cocultures with *H. bilis*. Upregulation of ECH1 could increase the biosynthesis of fatty acid metabolic intermediates required for the maintenance of cell membrane stability under the stress exerted by *H. bilis*. It also suggested mitochondrial stress and hepatic lipid oxidation, both of which have been linked to liver cirrhosis [29]. Among the regulated proteins of the cured Huh7 cells cocultured with *H. bilis*, was the tryptophan 5-monooxygenase activation protein YWHAQ, that participates in signal transduction.

Several of the metabolic enzymes identified in the transfected Huh7 cells in response to *H. bilis* presence were not identified in the cured Huh7 cells from which the replicon has been eliminated by IFN-*α* treatment or in the previously studied parental Huh7 cells. This indicates a synergy between the activities of the replicon and *H. bilis* on the metabolic pathway of the hepatoma cells. Also, the difference in the modulated metabolic proteins between the parental and cured Huh7 cells suggests the effect of IFN-*α* treatment on the cured cells and/or a permanent effect of the virus on the cells such that it responds *H. bilis* differently from the parental cells; a future study in which an IFN-*α* Huh7 cell population is included as a control would serve to resolve this. Nonetheless, this result is in concordance with the study of [30] which showed differences in the response to HCV infection of naïve, transfected and cured Huh7 cells.

### 3.5. Stress Response Proteins

Similarity in the upregulation of the heat shock proteins and chaperones was observed in both transfected and cured Huh7 cells in response to *H. bilis*. Similar upregulation in both cells was observed with the thio-specific antioxidant proteins, although peroxiredoxin 3 isoform precursor and peroxiredoxin 4 were identified only in the cured cells. Oxidative stress response proteins were downregulated in the transfected cells, but were not identified among the regulated proteins of the cured Huh7 cells. Notably, the 2 thioredoxin proteins identified were modulated differently in both cells: thioredoxin 1 was downregulated in the cured cells while in the transfected cells, the endoplasmic reticulum thioredoxin superfamily member protein was upregulated.

The data indicated that *H. bilis* exerted stress on the mitochondria resulting in the regulation of mitochondrial-localised DNAK, CPN60, PRXD3, and SOD proteins. The upregulation of the endoplasmic-reticulum- (ER-) specific TXNDC12 suggested also that an ER stress was exerted on the transfected Huh7 cells. The differences in the modulation between the transfected- and cured Huh7 cells of stress response proteins indicated that the presence of the HCV-replicon affected the molecular response of the hepatocytes to *H. bilis*.

This difference in the responses to *H. bilis* of the two cells not containing HCV replicon (parent Huh7 and cured Huh7 cells) suggested a permanent alteration of some cellular pathways by the virus or by the treatment with IFN-*α*. Generally, during the infection of hepatocytes by HCV, the lipid peroxidation and ROS generated by the hepatic inflammation that is induced due to the release of proinflammatory cytokines [31] could cause breaks in cellular DNA or mutations in different genes [32–34]. These mutations may result in permanent changes in some cellular pathways even when the virus has been inactivated. Similarly, treatment with IFN-*α* could lead to changes in the Huh7 cells; for example, changes in the functions of cellular factors were induced in Huh7 cells following exposure to IFN-*α* [35]; also, alterations in some signaling pathways of hepatoma cells treated with IFN-*α* have been reported [36]. Thus, the observed differences in the molecular response of the parent and the cured Huh7 cells to *H. bilis* could be the result of changes in the cells caused by the alterations of cellular pathways by the virus and/or by exposure to IFN-*α*.

### 3.6. Protein Translation, Modification, and Degradation

This category covers proteins that participate in the synthesis, translation, proper folding of other proteins, and degradation of misfolded proteins. Given the number and pattern of modulation of identified proteins in this category compared to other categories (overall total number of modulated proteins = 21. Total from transfected cells = 12: upregulated = 9, downregulated = 3. Total from cured cells = 13: upregulated = 11 and downregulated = 2), it is reasonable to infer that the presence of *H. bilis *caused major perturbation in the protein translation, modification, and degradation machineries of the cell. This becomes more apparent when compared with our data on the effect of the bacterium on the parent Huh7 cells in which all 7 modulated proteins were downregulated (data submitted elsewhere).

The upregulation of elongation initiation factor EIFA in the transfected cells and EIF5A in both the transfected- and cured Huh7 cells suggested overall increase in protein synthesis in both cells. The upregulation in both cell types of the prefolding subunit 2, PFDN2, which also participates in protein production by ensuring that newly synthesized polypeptides fold correctly through binding and stabilizing them, supports the notion that protein synthesis was upregulated by both cells in response to *H. bilis*. In the cured Huh7 cells, the human pre-mRNA splicing factor, SF2, was also upregulated, but the heterogeneous nuclear ribonucleoprotein C (HNRP) and mitochondrial ribosomal protein, MRPL17, which also functions in the stabilization of newly synthesized polypeptides, were downregulated. Thus, the downregulation of both HNRP and MRPL17 is counter intuitive to the notion of increased protein synthesis of the cells in response to *H. bilis*. Besides the functions of HNRPC and MRPL17 in stabilizing newly synthesized polypeptides and ensuring proper folding of newly translated proteins [37], both proteins are also involved in cellular apoptotic pathways [37–40]. Their downregulation would therefore affect the normal programmed death of stressed and diseased cells, which could induce cell changes leading to the emergence of mutated cells. Also counter intuitive to increased protein production in response to the bacteria was the downregulation of the translation elongation factors, EEF1D and EEF2D, in the cured Huh7 cells indicating that the presence of the bacterium affected the normal protein expression of the cell.

The effect of the bacterium in the normal protein expression of the cells could result in accumulation of misfolded and/or mutated proteins. This could explain the apparent activation by the cells of the the ubiquitin system, which is primarily involved in protein degradation [41]. Proteins destined for degradation are tagged with the ubiquitin protein through a series of enzymatic steps initiated by an ubiquitin-activating enzyme E1. Following activation, the ubiquitin is transferred to the ubiquitin-conjugating enzyme E2, and finally to a target protein in a process facilitated by the E3 ligase. Specific selection for targets is accomplished by E2 and E3 proteins. UBE2N is a human mono-ubiquitinated form of ubiquitin-conjugating enzyme which promotes the polyubiquitination of specific targets that modulate the activity of various cellular processes including DNA repair, mitotic progression, and nuclear factor-kappaB signaling [42]. Among the various types of E3 proteins SKP1 is a component of the SCF (SKP11/Culin/F-box protein) ligases. Ubiquilins function as shuttle vectors to deliver ubiquitinated proteins to the proteasomes in the endoplasmic reticulum for degradation. In transfected Huh7 cells, the upregulation of the E3 ligase SKP1, the ubiquilin UBQLN1, and the proteasome proteins PSMA2, PSMA3 and PSMA4 indicated an activation of the ubiquitin system, although UBE2N was downregulated suggesting a downregulation of the ubiquitin system at the point of ubiquitin conjugation. However, other conjugases such as UBE2D1, which are specifically associated with the SCF family of ligases, could salvage the system at the point of ubiquitin conjugation.

Further evidence of the bacteria effect on the ubiquitin pathway was the downregulation of ubiquitin carboxyl-terminal hydrolase enzymes, UCHL1, and UCHL3 in both transfected and cured cells. In addition, upregulation in both cells of cathepsin D, (CTSD), an apoptotic regulatory enzyme [43], whose upregulation has also been associated with HCC [44], indicate the perturbation of the apoptotic pathway and suggests a potential carcinogenic effects of the bacterium. 

Compared to the cured Huh7 cells, the transfected Huh7 cells modulated the expression of a larger number of proteins involved in protein degradation pathways, probably reflecting that *H. bilis* induced the generation of more abnormal proteins in the latter cells. This would suggest an exertion of a greater stress by the bacterium on the transfected Huh7 cells relative to the cured Huh7 cells. Similarly to the cured Huh7 cells, the parent Huh7 cells modulated fewer proteins that participate in protein degradation pathways (Data not shown). Hence, in comparison, *H. bilis* appeared to have exerted greater stress on the transfected Huh7 cells.

### 3.7. Cell Proliferation and Structure

Dysregulation of cell proliferation represents a protumorigenic principle in human hepatocarcinogenesis. The transfected- and cured Huh7 cells modulated a good number of proteins involved in the regulation of cell proliferation and structure (Table 1). Vimentin (VIM) is a cytoplasmic intermediate filament characteristic of mesenchymal cells usually not expressed in epithelial cells [45], but was upregulated by both the transfected- and cured Huh7 cell lines in response to *H. bilis*. Gilles and co-workers showed that the atypical expression of VIM in epithelial cancer cells might be associated with local invasiveness and metastasis potential [46]. The overexpression of VIM and its relation to tumor metastasis have been reported in several carcinomas [47–49], and its expression in the various cancer cells was essential to the successive shape change through the interaction with actin and other intermediate filaments [50]. Thus, the upregulation of VIM by both the transfected- and cured Huh7 cells may be correlated to the hummingbird morphology, and also indicates a carcinogenic effect of *H. bilis* on the cell lines.

Related to the regulation of cell structure, cytoskeletal proteins that control microfilament and microtubules were found among the modulated proteins of the transfected- and cured Huh7 cells. In both cell lines, beta-actin (ACTB) was upregulated. The highly conserved ubiquitous ACTB is regarded as a housekeeping gene involved in the formation of filaments that are a major component of the cytoskeleton, and participates in cell motility, structure, and integrity. Its upregulation by both Huh7 cells therefore shows that *H. bilis* affected basic cellular functions including cell morphogenesis. Upregulation of ACTB has also been reported in HCV-induced HCC [51] as well as in other cancer types [52, 53], suggesting a carcinogenic effect of *H. bilis.* An actin related protein 2/3 complex subunit 5 (ARPC5) was also upregulated in the transfected Huh7 cells but was downregulated by the cured Huh7 cells, and tubulin beta (TUBB) was downregulated in the transfected Huh7 cells but was upregulated in the cured Huh7 cells. The modulation of cytoskeletal proteins by the transfected- and cured Huh7 cells suggested a response to cytoskeletal stress that could disrupt the normal functioning of Huh7 cells and may result in the cells losing their ability to regulate shape and volume. This stress can promote abnormalities in cell morphology that lead to cell death, and help to explain the observed hummingbird morphology and decrease in cell proliferation observed in the Huh7-derived cell lines cocultured with *H. bilis*.

 Further, the modulation of TUBB indicated that the bacterium affected gap junction formations in both Huh7-derived cell lines. Gap junctions are connections between cytoplasm of two adjacent cells that make exchange of molecules and ions possible [54], and these are regulated by TUBB. In addition, TUBB is the building block of microtubules, which form structural cytoskeleton. Hence its up and downregulation in the cured- and transfected Huh7 cells respectively suggested a structural change in the presence of *H. bilis.* Similarly, modulation of ARPC5, a complex of the actin-related proteins ARP2 and ARP3 able to regulate actin polymerization [55], is another molecular event that could contribute to the change in morphology of the hepatoma cells in response to *H. bilis*.

### 3.8. Tumour-Related Proteins

Proteins that are associated with tumourigenesis were identified among those regulated in both the transfected- and cured Huh7 cells cocultured with *H. bilis*. In the former, transformation upregulated nuclear protein (HNRNP-K) was upregulated, together with tumour protein D52-like 2 isoform f (TPD52); C-MYC binding protein (MM1) was however downregulated in response to *H. bilis*. In cured Huh7 cells, hepatoma-derived growth factor (HDGF), rho GDP dissociation inhibitor alpha (GDI) and prohibitin (PHB) were upregulated, but B23 nucleophosmin (NPM) and DJ-1 protein (DJ-1) were downregulated in response to *H. bilis*.

HNRNP-K is highly upregulated in transformed cells. Its specific role in cell transformation is still largely unclear, but it is thought to be involved in cell cycle progression [56]. TPD52-like proteins are small coiled-coil motif bearing proteins originally identified through their elevated expression level in human breast carcinoma. It plays a role in calcium-mediated signal transduction and cell proliferation [57, 58]. TPD52 also regulates mitosis and facilitates membrane tethering and fusion through binding integral membrane and membrane-associated proteins, and its deregulated expression may affect adversely cell division and proliferation [59]. The protein is overexpressed in multiple human cancers [60, 61]. Its downregulation in the transfected Huh7 cells could contribute to the observed decrease in cell proliferation in the presence of *H. bilis*, and also suggested non-tumourigenic effects of *H. bilis*. The binding protein, MM1 is a pre-folding protein that binds to newly synthesized cellular myelocytomatosis oncoprotein (C-MYC), stabilizing and allowing it to fold correctly [62]. Its downregulation by the transfected Huh7 cells suggested that C-MYC too may be downregulated further indicating a non-tumourigenic effect of *H.bilis*.

In the cured Huh7 cell line, GDI, HDGF and PHB were upregulated, and NPM and DJ-1 were downregulated. GDI belongs to the family of Rho GDP dissociation inhibitors that include RhoGDI, D4-GDI, and RhoGDI-3 [63]. They regulate the reorganization of the actin cytoskeleton and the integrity of associated integrin adhesion complexes [63]. GDI is expressed ubiquitously and is directly involved in cell adhesion. In breast cancer tissue, upregulation of GDI has been linked to the induction of Raf-1, a protein serine-threonine kinase, which plays a role in cell growth, proliferation, and cell survival [64]. Thus, the upregulation of GDI could be a tumourigenic response of the cured Huh7 cell line to *H. bilis*. HDGF is an acidic heparin-binding protein originally isolated from the cultured media of the Huh7 cell line [65]. It is expressed ubiquitously in normal tissue and in tumour cell lines [66, 67]. The protein has mitogenic effect because recombinant HDGF has been shown to stimulate the growth of a variety of cells including hepatoma cells [65, 68, 69]. Studies have indicated that HDGF participates in cellular proliferation and differentiation [66, 70], and its increased expression has been associated with poorly differentiated HCC cell lines and tumour progression in HCC specimens where it is thought to enhance the unregulated growth or recurrence of hepatoma cells [71]. The downregulation of HDGF by the cured Huh7 cells in cocultures with *H. bilis* therefore did not indicate a cancer-promoting response of the cell line to the presence of the bacterium.

Prohibitin-PHB is localized in the mitochondria, is essential for normal mitochondrial development, and plays a role in tumour suppression, proliferation, cell-cycle progression, and apoptosis [72]. PHB also co-localises with retinoblastoma protein (pRb) and P53, which are known tumour suppressor proteins, and interacts with the transcription factor E2F in various cell lines [73, 74]. These observations indicate that PHB blocks cell proliferation and acts as tumour suppressor by cell-cycle arrest via the repression of E2F-mediated transcription [72, 75]. Dysregulation of PHB has not been documented in HCC, but its upregulated expression has been reported in other human cancer tissues and cell lines [76, 77]. The upregulation of PHB suggested a tumour-suppression effect of *H. bilis* on the cured Huh7 cell line, and could account for the decrease of Huh7 cells proliferation in the presence of the bacterium.

Many studies have suggested that NPM may be involved in cancer pathogenesis [78], however, its physiological function in tumourigenesis is still controversial since this protein has been ascribed both tumour suppressive and oncogenic functions [78]. Its downregulation in cured Huh7 cells would reflect a cancer-promoting effect of the bacterium. Functionally, DJ-1 has been implicated in fertilization [79], the regulation of androgen receptor signaling [80], and oxidative stress [81]. In addition, DJ-1 is involved in apoptosis [82] and cancer [83]. Its role in cancer is thought to involve the suppression of the tumour suppressor PTEN which in turn affects the function of the protein kinase B (PKB/AKT), a survival kinase [84]. The downregulation of DJ-1 would enhance the suppression of PKB/AKT by PTEN leading to a decrease in cell survival, a phenomenon that may help to explain the observed reduction in cell proliferation of the cured Huh7 cells in the presence of *H. bilis* and suggest a nontumourigenic effect of the bacterium on the cured hepatoma cell line.

**Figure 3:**
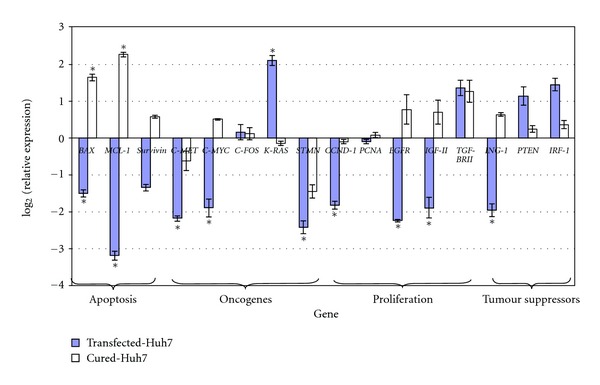
Relative expression of HCC-related genes in transfected- & cured Huh7 cells in response to *H. bilis*. Values are presented as log_2_ of the relative expression and represent the mean relative expression derived from biological triplicates. *P < 0.05.

Additional investigations of the potential tumourigenic effect of *H. bilis* on the transfected- and cured Huh7 cell lines were carried out by studying the differential expression of 16 HCC-related genes employing qRT-PCR (Figure 3).

Overall, the changes in the transcription of these HCC-related genes differed between transfected- and cured Huh7 cells exposed to *H. bilis*. The genes *BAX* and *MCL-1* were downregulated in the transfected Huh7 cells but were upregulated in the cured Huh7 cells in the presence of *H. bilis*. Both genes belong to the B-cell lymphoma 2 family of apoptotic proteins, *BAX* is proapoptotic while *MCL-1* is antiapoptotic, and their dysregulation has been associated with HCC [85, 86]. The downregulation of *BAX* in the transfected Huh7 cells would enhance cell survival; in contrast, the downregulation of *MCL-1* would enhance cell death. On the other hand, the upregulation of *BAX* and *MCL-1* by the cured Huh7 cells would enhance cell death and cell survival, respectively. Thus, the modulation of the expression of these two genes suggested a complex regulation of apoptosis in response to the presence of *H. bilis* which was different in both types of cells.

The oncogene *KI-RAS* was upregulated while *C-MET*, *C-MYC*, and *STMN* were downregulated by the transfected Huh7 cells. *KI-RAS* belongs to the *RAS* family of GTP-binding proteins and signals through the RAS/RAF/MEK/ERK pathway to activate the downstream transcription factors including NK-*κ*B, CREB, AP-1, and C-MYC [87], all of which have roles in regulating proliferation and apoptosis [88]. C-MET is a hepatocyte growth factor receptor encoding tyrosine-kinase activity. C-MET also signals via the RAS/RAF/MEK/ERK as well as the P13/AKT/PTEN pathways [89]. In HCC, C-MET is an essential factor in the process of migration and invasion of hepatocarcinoma cells [90]. Thus, the downregulation of these effector oncogenes, regardless of the upregulation of *KI-RAS*, is consistent with the proposition that *H. bilis* promoted anticancer activity in the transfected Huh7 cells.

The cell proliferation regulators *CCND-1*, *EGFR* and *IGF-II* were downregulated in the transfected Huh7 cells. EGFR is a receptor for the transforming growth factor alpha (TGF-*α*), a mitogenic growth factor required to trigger the proliferative state. Expression of EGFR is associated with HCC [91]; its inhibition induces growth arrest and apoptosis in HCC cell lines [92] and prevents HCC development in rats [93]. Thus, its downregulation would promote the cell death of the transfected Huh7 cells. In addition, EGFR together with IGF-II signal through the P13K/AKT/PTEN and RAS/RAF/MEK/ERK pathways and promote the downstream activation of genes including the *CCND1*, thus, the downregulation of *EGFR* and *IGF-II* suggested an antitumour response by the human cells and correlated with the downregulation of *CCND1* by the transfected Huh7 cells in response to *H. bilis*. CCND1 acts in concert with cyclin-dependent kinase to phosphorylate and activate the retinoblastoma protein (pRB), which is involved in cell proliferation. Upregulation of *CCND1* has been reported in human and mice HCC tissues [94], thus, its downregulation suggested also a nontumourigenic effect of *H. bilis* on the transfected Huh7 cells. In contrast, the downregulation of the tumour suppressor gene *ING1*, whose product is a nuclear protein that physically interacts with the tumor suppressor protein TP53 in the p53-signaling pathway [95], and can induce cell growth arrest and apoptosis, suggested a tumourigenic effects of *H. bilis* on the transfected Huh7 cells.

## 4. Conclusion

Taken together, the differential expression of some tumour-related proteins and HCC-associated genes provided evidence for the carcinogenic effects of H. bilis on the Huh7-derived cell lines, although the expression pattern of other proteins and genes did not support this conclusion. This apparent contradiction may be explained by the fact that hepatocarcinogenesis involves multiple factors and pathways and are dependent on the carcinogen. Thus, it is possible that *Helicobacter* spp. could initiate dysplasia in hepatocytes through several processes some of which may not involve known classical pathways in HCC development. The result further shows that *H. bilis* exerted multiple stresses on both the transfected and cured Huh7 cell lines, which appeared to have affected the function of the mitochondria and endoplasmic reticulum. At the molecular level, the transfected Huh7 cell line expressed genes which indicated that the cell line was more susceptible to the effect of the bacterium than to the cured Huh7 cell line. Thus, it is possible that *H. bilis* could potentiate the development from dysplasia to neoplasia of the liver cells in the background of HCV-infection. The similarities between the transfected and cured Huh7 cell lines in the modulation of proteins belonging to biological functional categories, some of which are implicated in cell necrosis, revealed some common response of both cell lines to the presence of *H. bilis* regardless of the presence of the HCV-replicon in the transfected-cells, and suggested that the bacterium could initiate preneoplastic processes in these cell lines.

## Figures and Tables

**Table 1:** Proteins of transfected and cured Huh7 cells where expression was modulated in cocultures with *H. bilis* and identified by LC/MS-MS.

	n-Ffold (±SEM)^b^		Gene symbol	Function^c^	Spot no.		Matching score^d^	
Protein^a^	T-Huh7^e^	C-Huh7^f^			T-Huh7	C-Huh7	T-Huh7	C-Huh7
				Regulation of cell proliferation and structure				

Vimentin^∗^	5 ± 0.01	3.6 ± 0.02	*VIM*		17	11	2228	1827
Vimentin^∗^	3 ± 0.03	2.3 ± 0.1	*VIM*		18	12	2166	1490
Tubulin beta	0.4 ± 0.3	2.7 ± 0.3	*TUBB*		14	21	112	962
Tubulin beta^∗^	NI	2.4 ± 0.5	*TUBB*		—	45	—	341
Transmembrane protein 4	4.5 ± 0.04	NI	*CNPY2*		51	—	338	—
Stomatin-like 2	NI	2.4 ± 0.3	*SLP2*	Cytoskeleton and structural organization	—	10	—	1272
Beta actin	NI	2.35 ± 0.04	*ACTB*		—	24	—	560
Mutant beta-actin^∗^	2.7 ± 0.01	NI	*ACTB*		20	—	166	—
Mutant beta-actin^∗^	2.1 ± 0.3	NI	*ACTB*		38	—	161	—
Actin related protein 2/3 complex, subunit 5-like	2.2 ± 0.1	0.4 ± 0.1	*ARPC5*		47	40	181	422
Stathmin 1	0.3 ± 0.1	3.2 ± 0.02	*STMN1*		46	39	536	322

Integrin beta 4 binding protein isoform a	0.3 ± 0.01	NI	*ITGB4*	Cell-matrix adhesion	33	—	139	—
Ran-binding protein 1	0.4 ± 0.1	NI	*RAN-BP1*		31	—	147	—

				Metabolism and Biosynthesis enzymes				

Malate dehydrogenase	0.4 ± 0.1	NI	*MDHA*		10	—	451	—
Enolase^∗^	2.1 ± 0.3	NI	*ENO*		23	—	782	—
Enolase^∗^	4.2 ± 0.02	NI	*ENO*	Energy metabolism	53	—	800	—
Triosephosphate isomerase 1	2.1 ± 0.3	2.6 ± 0.3	*TIM*		26	34	1115	1210
ATP synthase beta sub unit	3.5 ± 0.2	2.4 ± 0.2	*ATP5B*		9	36	75	423
ATP synthase, H^+^ transporting mitochondrial F0 complex sub unit d isoform a	2.1 ± 0.3	NI	*ATP5H*		35	—	139	—

UV excision repair protein RAD23 homolog B	2.1 ± 0.1	NI	*RAD23B*	Nucleic acid metabolism (DNA repair)	16	—	889	—
Enoyl coenzyme A hydratase 1, peroxisomal	4.8 ± 0.01	NI	*ECH1*	Fatty acid metabolism	24	—	549	—
Tryptophan 5-monooxygenase activation protein	NI	2.7 ± 0.3	*YWHAQ*	Amino acid metabolism	—	27	—	609
Selenophosphate synthetase	NI	4.3 ± 0.02	*SEPHS1*		—	8	—	584
Pyrophosphatase 1	NI	3.7 ± 0.01	*PPA1*	Nucleotide metabolism	—	22	—	201

				Protein translation, Modification, and Degredation				

Heterogeneous nuclear ribonucleoprotein	NI	3.4 ± 0.03	*HNRP*	RNA processing	—	6	—	870

Valosin-containing protein^∗^	0.4 ± 0.1	NI	*VCP*		1	—	3218	—
Valosin-containing protein^∗^	0.2 ± 0.1	NI	*VCP*		2	—	2178	—
Valosin-containing protein^∗^	0.4 ± 0.1	NI	*VCP*		3	—	1318	—
Prefoldin subunit 2	3.8 ± 0.02	2.9 ± 0.1	*PFDN2*		43	38	378	299
Cathepsin D preprotein	2.7 ± 0.3	2.2 ± 0.3	*CTSD*		27	9	370	615
Ubiquitin-conjugating enzyme E2N	0.4 ± 0.3	NI	*UBE2N*		44	—	230	—
Ubiquilin 1^∗^	2.4 ± 0.01	NI	*UBQLN1*		12	—	116	—
Ubiquilin 1^∗^	2.1 ± 0.3	NI	*UBQLN1*	Protein degradation	13	—	602	—
Ubiquitin carboxy-terminal hydrolase L1	0.3 ± 0.1	NI	*UCHL1*		30	—	230	—
Ubiquitin carboxyl-terminal esterase L3	NI	2.5 ± 0.06	*UCHL3*		—	29	—	320
S-phase kinase-associated protein 1A isoform b	3.2 ± 0.05	NI	*SKP1*		42	—	124	—
Proteasome alpha 3 subunit isoform 1	2.1 ± 0.3	4.1 ± 0.01	*PSMA3*		52	26	63	71
Proteasome 26S non-ATPase subunit 4	2.5 ± 0.1	NI	*PSMA4*		15	—	599	—
Prolyl 4-hydroxylase, alpha 1 subunit isoform 2 precursor	NI	2.5 ± 0.03	*P4HA1*		—	5	—	888
Heme binding protein 2	2.8 ± 0.01	NI	*HEBP2*		32	—	107	—
Small glutamine-rich tetratricopeptide	NI	0.1 ± 0.01	*SGTA*		—	20	—	403

Eukaryotic translation initiation factor 3	3.0 ± 0.02	NI	*EIF3A*		22	—	994	—
Eukaryotic translation initiation factor 5A^∗^	2.7 ± 0.1	2.3 ± 0.05	*EIF5A*		49	44	542	253
Eukaryotic translation initiation factor 5A^∗^	2.8 ± 0.1	NI	*EIF5A*	Protein translation, modification, and elongation activity	50	—	147	—
Eukaryotic translation elongation factor 1 delta isoform 2	NI	2.2 ± 0.4	*EEF2D*		—	18	—	627
Eukaryotic translation elongation factor 1 delta isoform 1	NI	0.4 ± 0.1	*EEF1D*		—	19	—	565
Human pre-mRNA splicing factor	NI	2.1 ± 0.2	*SF2*		—	37	—	61

Mitochondrial ribosomal protein L7L12^∗^	NI	2.1 ± 0.4	*MRPL17*	Ribosomal structure, and biogenesis	—	42	—	164
Mitochondrial ribosomal protein L7L12^∗^	NI	2.4 ± 0.1	*MRPL17*		—	43	—	86

				Regulation of transcription				

Basic transcription factor 3-like 4	0.4 ± 0.5	NI	*BTF3L4*	DNA binding	36	—	106	—
WD repeat domin 77	NI	2.5 ± 0.03	*WDR*	Coordination of multiprotein complex assemblies	—	14	—	166

				Stress response				

Mitochondria heat shock protein 75^∗^	2.3 ± 0.2	2.7 ± 0.1	*DnaK*		4	1	1817	3016
Mitochondria heat shock protein 75^∗^	2.7 ± 0.5	3.1 ± 0.1	*DnaK*		5	2	1817	2383
Heat shock 70 kDa protein 8 isoform 1^∗^	2.9 ± 0.1	2.3 ± 0.3	*HSPA8*	Heat shock proteins and chaperones	6	3	2463	2383
Heat shock 70 kDa protein 8 isoform 1^∗^	2.6 ± 0.1	2.7 ± 0.1	*HSPA8*		7	4	1822	1792
Chaperonin	2.3 ± 0.2	NI	*Cpn60*		21	—	1361	—
T-complex polypeptide 1 (chaperonin)	0.15 ± 0.01	NI	*TCP1*		8	—	336	—

Superoxide dismutase copper chaperone	0.35 ± 0.2	NI	*HMA*	Oxidative stress response	25	—	203	—
Cu/Zn superoxide dismutase	0.2 ± 0.03	NI	*CuZnSOD*		37	—	69	—

Peroxiredoxin 2 isoform a	2.4 ± 0.05	2.7 ± 0.2	*PRDX2*		34	35	564	78
Peroxiredoxin 3 isoform a precursor	NI	2.5 ± 0.1	*PRDX3*	Thio-specific antioxidant protein	—	31	—	179
Peroxiredoxin 4	NI	3.2 ± 0.05	*PRDX4*		—	30	—	782

Thioredoxin-like 1	NI	0.37 ± 0.1	*TXNL1*	Oxidoreductase activity	—	—	17	758
Endoplasmic reticulum thioredoxin superfamily member	4.7 ± 0.01	NI	*TXNDC12*		48	—	112	—

CGI-150 protein	NI	2.1 ± 0.1	*CGI*	Response to toxin	—	23	—	521

				Signal transduction and transport				

Progesterone receptor membrane component 1^∗^	0.3 ± 0.2	NI	*PGRMC1*		40	—	322	—
Progesterone receptor membrane component 1^∗^	0.35 ± 0.4	NI		Signal transduction	41	—	294	—
Phospholipase C-alpha	0.3 ± 0.03	NI	*PLC*α**		19	—	227	—
Glia maturation factor, beta	NI	2.8 ± 0.01	*GMFB*		—	41	—	233

Sorcine isoform a	3.1 ± 0.01	NI	*SRI*	Transmembrane transporter protein	39	—	320	—
Calcium-binding mitochondrial carrier	NI	0.2 ± 0.01	*EFH*		—	7	—	242

				Tumour-related				

Tumor protein D52-like 2 isoform f	3.7 ± 0.03	NI	*TPD52*	Tumorigenesis	29	—	63	—
Hepatoma-derived growth factor	NI	2.9 ± 0.01	*HDGF*	Cellular proliferation and differentiation	—	13	—	65
Transformation upregulated nuclear protein	4.2 ± 0.01	NI	*HNRNP-K*		54	—	116	—

Rho GDP dissociation inhibitor alpha	NI	2.3 ± 0.2	*GDI*		—	28	—	412
C-myc binding protein	0.3 ± 0.03	NI	*MM1*		45	—	159	—
RAS related protein	NI	3.1 ± 0.1	*RAB33*		—	33	—	147
Immunity-related GTPase	3.0 ± 0.3	NI	*IRGQ*	Oncogene related	11	—	125	—
B23 nucleophosmin^∗^	NI	2.9 ± 0.1	*NPM*		—	15	—	1065
B23 nucleophosmin^∗^	NI	3.2 ± 0.1	*NPM*		—	16	—	1091
DJ-1 protein	NI	0.2 ± 0.01	*DJ-1*		—	32	—	429

Prohibitin	NI	3.2 ± 0.1	*PHB*	Negative regulator of apoptosis	—	25	—	59

^a^ Proteins were identified by LC-MS/MS and MASCOT searches of the NCBI nr database. ^b^Differential expression by at least 2-fold level of proteins of transfected or cured Huh7 cells exposed to sublethal concentration of *H. bilis* initial inoculum density of 10^3^ cfu/mL; an n-fold value of >2 indicates upregulation, while a value of <0.5 indicates downregulation. ^c^Proteins were classified into functional categories based on the PANTHER, KEGG, Swiss-Prot and NCBI databases, and by reference to the published literature. ^d^Proteins with peptide matching score of >49 were considered significant for identification. ^∗^Protein isoforms. ^e^Transfected Huh7 cells; ^f^Cured Huh7 cells.
